# Effects of Direct Current Administration on Hyperhidrosis Disease Severity Scale in Patients with Axillary Hyperhidrosis

**DOI:** 10.1155/2019/3232015

**Published:** 2019-10-31

**Authors:** Serpil Şener, Yunus Karakoç

**Affiliations:** ^1^Department of Dermatology, Inonu University, Faculty of Medicine, Malatya, Turkey; ^2^Department of Biophysics, Health Sciences University, Faculty of Medicine, Istanbul, Turkey

## Abstract

**Background:**

Focal hyperhidrosis mostly affects the axillae, hands, feet, and face. For the management, several techniques are used. The aim of this study was to investigate the effects of direct current administration on the hyperhidrosis disease severity scale (HDSS) in patients with axillary hyperhidrosis that have various HDSS scores.

**Design and Setting:**

Original article, University Hospital.

**Methods:**

Sixty patients with primary axillary hyperhidrosis were inquired about the HDSS scores and the scores were noted at the onset and at the end of the 10th application. One month after the last session, HDSS scores were also inquired. At the end of 1-month follow-up, the patients whose HDSS scores rose after the 10th session were accepted as nonresponder. For the current delivery, a new iontophoresis application module (Sweat CureR) designed by Dr. Karakoc was used.

**Results:**

Direct current application decreased axillary sweat intensity by 70% at both sides, and lowered the HDSS by about 1.5 degree. Major reduction in sweat intensity was in the patients with low HDSS scores (75%). Negative correlation was found between initial HDSS scores and median values of decreased sweat intensity (*r* = −0.317, *p* = 0.022). Minimal temporary side effects including skin irritation and one or more vesicle formation were inspected in 29 patients and, the permanent punctual pigmentation was observed only in one patient.

**Conclusions:**

Decrease in axillary hyperhidrosis is satisfactory for these patients. Since iontophoresis application has beneficial effect and minimal side effects, it should be recommended to the patients before advanced management or surgical techniques.

## 1. Introduction

Hyperhidrosis, a disorder of excessive sweat, may be focal, involving specific areas of the body, or generalized, involving the entire body. Focal hyperhidrosis most commonly affects the axillae, hands, feet, and face [1]. Altogether, some 3% of the population suffers from hyperhidrosis, and 51% of these from focal axillary hyperhidrosis [2]. Axillary hyperhidrosis is most common after the onset of puberty, when the axillary apocrine glands begin proliferation [3]. For the management of focal hyperhidrosis several techniques are used [4–7]. Efficacy of direct current administration namely iontophoresis had been proven for palmoplantar hyperhidrosis in several studies [8–12]. However, this technique is not recommended for axillary hyperhidrosis [1]. For patients with moderate to severe hyperhidrosis, the committee recommends starting treatment with topical aluminum chloride, and, if ineffective, trying iontophoresis or botulinum toxin A injections. Further interventions, such as surgery, should be reserved for patients who do not respond to less invasive interventions [2, 13].

A subjective scale introduced by the Canadia Hyperhidrosis Advisory Committee, the Hyperhidrosis Disease Severity Scale (HDSS), allows patients to characterize the severity of their hyperhidrosis on a scale from 1 to 4. This scale is useful for assessment of relative improvement with therapy [7].

## 2. Objective

The aim of this study was to investigate the effects of direct current administration on the hyperhidrosis disease severity scale (HDSS) in patients with axillary hyperhidrosis that have various HDSS scores.

## 3. Patients and Methods

**Figure 1:**
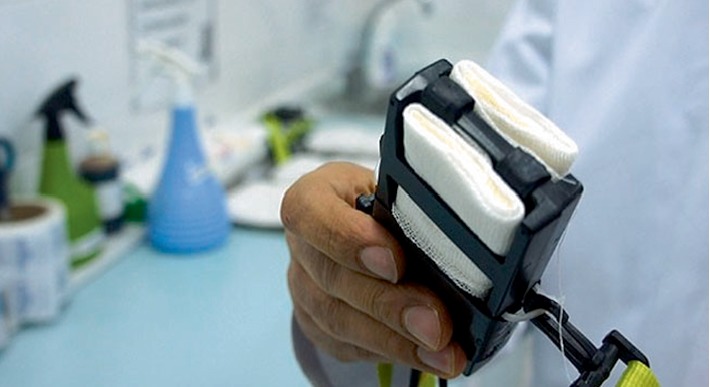
New iontophoresis application module for axillary hyperhidrosis designed by Karakoc (Sweat CureR, Patent Number: TR 2012 10825 B).

**Figure 2:**
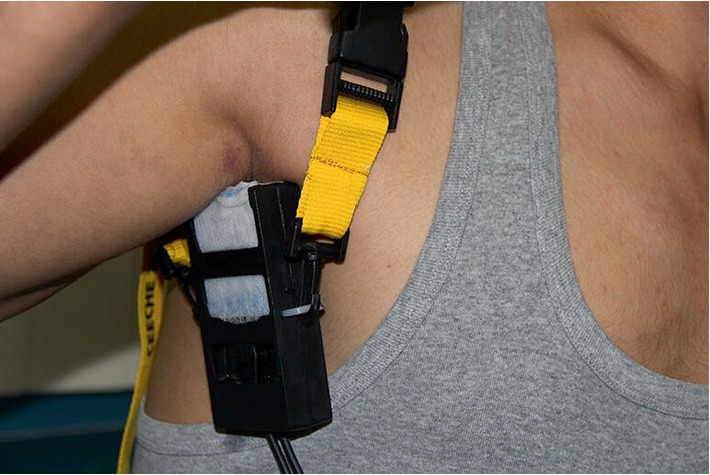
Illustration of iontophoresis application module to treat axillary hyperhidrosis. Arm overlies the module during operation.

After the approval of our study protocol by local ethic committee (ethics committee number 2011/12), 60 patients with axillary hyperhidrosis were enrolled in the study. Dermatology and endocrinology departments examined patients and evaluated whether their complaints were about primary or secondary hyperhidrosis and, the selected patients with primary axillary hyperhidrosis were directed to iontophoresis application. Having examined axillary areas of patients against to contraindications of iontophoresis, 10 sessions were planned for each one and signed informed consents were taken. Demographic parameters and clinical features of patients were noted. The Hyperhidrosis Disease Severity Scale (HDSS) was chosen to measure disease severity in the proposed treatment algorithms. It is a disease-specific scale for hyperhidrosis that provides a qualitative measure of the severity of the patient's condition based on how it affects daily activities [1]. Patients were inquired about the HDSS scores and the scores were noted at the onset and at the end of the 10^th^ application. One month after the last session HDSS scores were also inquired by calling the patients. At the end of 1-month follow-up, the patients whose HDSS scores rose in comparison with the 10^th^ session were accepted as non-responder. For the axillary iontophoresis applications, we used a new iontophoresis application module designed by Dr. Karakoc (SweatCureR; Patent Number: TR 2012 10825 B) that delivers the direct current to one of the axillary areas in connection to the electrotherapetic current stimulator (Figures 1 and 2). Iontophoresis applications had performed at 1st, 3rd, and 5th days of week days for 10 sessions within one month. Iontophoresis application took 20 min for one side and, one session was totally 40 min for both sides. Current density was adjusted to tolerable limits for each patient and changed with patient's comfort but restricted by a maximum 7 mA. This schedule was slightly changed for some patients who had skin irritation after any session.

Statistical analyses were performed using Statistical Package for the Social Sciences (SPSS) for Windows. Number and percent values of the patients were calculated for each demographic parameter and category and, median value of decreasing rate (%) in sweat intensity were determined according to HDSS categories. Pearson correlation test was used to investigate a relationship between HDSS and sweat intensity reduction. Decrease in HDSS score was correlated with the reduction of sweat intensity and, 80% reduction was accepted 2 degrees fall while 50% reduction was 1 degree fall in HDSS scores.

## 4. Results

Demographic features and clinical history of patients are summarized in Table 1. The mean age of patients was found 29.3 ± 9.3 year. The mean initial HDSS scores were similar for both gender and found as 3.7 ± 0.6 and 3.6 ± 0.6 for men and women, respectively. Majority of the patients (88.1%) completed 10 sessions and declared the reasonable reduction in sweat intensity at the end of the 10th session and one month after the last session (Table 2). One patient did not complete 10 sessions and was excluded from the study. We demonstrated that direct current application decreased axillary sweat intensity by about 70% at both sides, and lowered the HDSS by about 1.5 degree. Most reduction in sweat intensity was in the patients with low HDSS scores (75%) (Table 3). A negative correlation was found between initial HDSS scores and median values of decreased sweat intensity (*r* = −0.317, *p* = 0.022). Minimal temporary side effects including skin irritation and one or more vesicle formation were inspected in 29 patients during the treatment period and, the permanent punctual pigmentation on the armpit was observed only in one patient.

## 5. Discussion

Sweating is a physiological and vital process. The basic distinction is made between two types of sweating: thermoregulatory and emotional sweating. Most of the sweat glands are of the eccrine type. They produce a thin secretion that is hypotonic to plasma. Eccrine sweat glands are distributed all over the body; their highest density is in the axillary region, on the palms of the hands, and on the soles of the feet [2, 14].

Majority of minimal temporary side effects were healed by using topical dexpanthenol cream without any defects. During iontophoresis applications, only vesicles or irritated points on the armpit were covered with parafilm coated silk adhesive plasters in order to prevent further irritation and eliminate pain sensation.

Of course, quantitative measurements are superlative than the subjective evaluation of sweat intensity with HDSS scores declared by the patients. However, when sweat intensity is measured at any time, it may not reflect the patient's relief or severity of disease due to fluctuations of sweat production throughout day time. Hence, HDSS can be reliable parameter for the self-assessment of patients in their daily life.

Unfortunately, iontophoresis is not recommended for axillary hyperhidrosis because of ineffectiveness and difficulties on the axillary area [2]. Thomas et al. showed that only 4 of the 17 patients (24%) deemed the treatment successful and reported an adequate clinical response, while 6 (35%) reported clinically inadequate improvement, and 7 (41%) reported no change or worsening of their symptoms [3]. Conversely, if this technique is used more correctly its effectiveness is nearly the same as the effectiveness for palmar hyperhidrosis. By using new iontophoresis application module (Sweat CureR) to deliver direct current to one side of axillary areas with a current stimulator, 88% of patients were treated successfully and reduction of sweat intensity was found at about 67%.

From our results, decreases in axillary hyperhidrosis and HDSS are satisfactory for the patients with axillary hyperhidrosis. Since this iontophoretic application has beneficial effect and minimal side effects, it should be recommended to the patients before advanced management or surgical techniques. Algorithms for the management of axillary hyperhidrosis must include this application for all levels of axillary hyperhidrosis after the topical aluminum chloride application.

## 6. Conclusion

It can be said that iontophoresis treatment is a safe and easy method for the treatment of axillary hyperhidrosis.

## Figures and Tables

**Table 1:** History of patients with axillary hyperhidrosis.

	Male (30)	Female (29)	Total (59)
Age (year)	29.3 ± 9.3	27.1 ± 8.9	31.5 ± 9.5
HDSS	3.7 ± 0.6	3.6 ± 0.6	3.7 ± 0.5
Palmar hyperhidrosis	18 (30.5%)	9	9
Plantar hyperhidrosis	17 (28.8%)	6	11
Familial hyperhidrosis	25 (42.4%)	11	14
Onset at childhood	19 (32.2%)	10	9
Onset at adolescent	7 (11.9%)	3	4
Herediary disease history	22 (37.3%)	10	12

**Table 2:** Effectiveness of iontophoresis application on axillary hyperhidrosis.

	Male	Female	Total
Responders	25	27	52 (88.1%)
Nonresponders	5	2	7 (11.9%)
Total	30	29	59 (100%)

**Table 3:** Effectiveness of iontophoresis application on HDSS scores.

Initial HDSS	*N*	Decrease in sweat intensity (median%)
2	3	75.0
3	10	72.5
4	39	65.0
Total	52	70.0

## Data Availability

The data used to support the findings of this study are available from the corresponding author upon request.
